# Disclosing *in utero* HIV/ARV exposure to the HIV-exposed uninfected adolescent: is it necessary?

**DOI:** 10.7448/IAS.19.1.21099

**Published:** 2016-10-13

**Authors:** Jennifer Jao, Rohan Hazra, Claude A Mellins, Robert H Remien, Elaine J Abrams

**Affiliations:** 1 Department of Medicine, Icahn School of Medicine at Mount Sinai, New York, NY, USA; 2 Department of Obstetrics, Gynecology and Reproductive Science, Icahn School of Medicine at Mount Sinai, New York, NY, USA; 3 Maternal and Pediatric Infectious Disease Branch, Eunice Kennedy Shriver National Institute of Child Health and Human Development, Bethesda, MD, USA; 4 HIV Center for Clinical and Behavioral Studies, New York State Psychiatric Institute and Columbia University, New York, NY, USA; 5 ICAP, Mailman School of Public Health and College of Physicians and Surgeons, Columbia University, New York, NY, USA

**Keywords:** HIV exposure, disclosure, *in utero*, antiretrovirals

## Abstract

**Introduction:**

The tremendous success of antiretroviral therapy has resulted in a diminishing population of perinatally HIV-infected children on the one hand and a mounting number of HIV-exposed uninfected (HEU) children on the other. As the oldest of these HEU children are reaching adolescence, questions have emerged surrounding the implications of HEU status disclosure to these adolescents. This article outlines the arguments for and against disclosure of a child's HEU status.

**Discussion:**

Disclosure of a child's HEU status, by definition, requires disclosure of maternal HIV status. It is necessary to weigh the benefits and harms which could occur with disclosure in each of the following domains: psychosocial impact, long-term physical health of the HEU individual and the public health impact. Does disclosure improve or worsen the psychological health of the HEU individual and extended family unit? Do present data on the long-term safety of *in utero* HIV/ARV exposure reveal potential health risks which merit disclosure to the HEU adolescent? What research and public health programmes or systems need to be in place to afford monitoring of HEU individuals and which, if any, of these require disclosure?

**Conclusions:**

At present, it is not clear that there is sufficient evidence on whether long-term adverse effects are associated with *in utero* HIV/ARV exposures, making it difficult to mandate universal disclosure. However, as more countries adopt electronic medical record systems, the HEU status of an individual should be an important piece of the health record which follows the infant not only through childhood and adolescence but also adulthood. Clinicians and researchers should continue to approach the dialogue around mother–child disclosure with sensitivity and a cogent consideration of the evolving risks and benefits as new information becomes available while also working to maintain documentation of an individual's perinatal HIV/ARV exposures as a vital part of his/her medical records. As more long-term adult safety data on *in utero* HIV/ARV exposures become available these decisions may become clearer, but at this time, they remain complex and multi-faceted.

## Introduction

With the widespread use of combination, antiretroviral therapy (ART) for the prevention of mother-to-child transmission (PMTCT) of HIV, vertical transmission rates have dwindled to <2% [1–3]. The tremendous success of PMTCT has resulted in a diminishing population of perinatally infected children on the one hand and a mounting number of HIV-exposed uninfected (HEU) children on the other. It is estimated that approximately 20% of all infants born in sub-Saharan Africa are born HEU [4].

Households comprising HIV-infected women and HEU children often face significant socio-economic stressors with limited healthcare access, high levels of perceived stigma and low levels of psycho-social support [5–7]. Disclosure of a child's HEU status, by definition, requires disclosure of maternal HIV status, and this may be difficult given the mother's right to privacy and concern for safety, particularly with continuing stigma around HIV. Disclosure of a mother's HIV status to her children has been low with rates ranging from 20 to 60% in the United States [8,9] and 40 to 50% in sub-Saharan Africa [5,10]. While much of the disclosure literature has focused on a mother's disclosure of her HIV status to her children, exploring potential benefits to a mother's own health [8,11], little has been published on the disclosure of a child's *in utero* exposure to HIV and antiretroviral medications (ARVs) and whether this has direct risks or benefits to the child's health [12].

The oldest of HEU children are now reaching adolescence and early adulthood, an important transition period often marked by concerns around diminished healthcare access and utilization [13]. As HEU adolescents transition from paediatric to adult healthcare, many assume responsibility for their own healthcare decisions during an already complex phase of cognitive, psychosocial and developmental changes. This responsibility requires knowledge of their medical history, which may include information about perinatal exposures such as exposure to *in utero* HIV/ARV. Today, we face unknowns regarding the long-term safety of intrauterine HIV/ARV exposure into adulthood and an increasingly large and aging population of HEU children. At the intersection of these issues, the landscape of HIV disclosure is beginning to consider not only benefits/harms for the mother and her family regarding disclosure of maternal HIV status but also benefits/harms for the child regarding disclosure of a child's perinatal HIV/ARV exposure status. Researchers evaluating the long-term safety of intrauterine HIV/ARV exposures through prospective cohort studies require long-term monitoring of HEU children into adulthood necessitating consent from such individuals when they turn 18, resulting in a need to disclose perinatal HIV/ARV exposures to the HEU participant. Clinicians assuming the healthcare of HEU adolescents may struggle with how to best monitor HEU patients in the setting of a lack of conclusive data on the long-term risks of intrauterine HIV/ARV exposures. HEU adolescents and their mothers may have competing desires for privacy due to persistent stigma and the need to avert other psychosocial harms. Adolescents transitioning to adult care may not be fully emotionally and mentally prepared to assume responsibility for their own health as this can be an unstable period of experimentation and individuation which supersedes desires to participate in consistent healthcare. In this article, we summarize the arguments for and against disclosure of intrauterine HIV/ARV exposure to HEU children/adolescents.

## Discussion

### Monitoring of HEU children: current guidelines

We begin our discussion with a related but separate question involving whether HEU children merit long-term monitoring, since the answer to this question has direct impact on whether disclosure of a child's intrauterine HIV/ARV exposure should occur. We systematically reviewed all English, French and Spanish articles identified in a PubMed/Medline database up to July 2016 on guidelines for the monitoring of HEU children and contacted several key in-country researchers leading surveillance and research initiatives in this area. While there is no consensus on the type of monitoring which should occur, several countries have developed systems and guidelines (Table 1). Wide variability in the recommended duration and intensity of longitudinal observation exists, perhaps due to the fact that this is a rapidly evolving area where emerging needs of HEU children are slowly rising to the forefront. Mechanisms of monitoring encompass registry/surveillance programmes and national research cohorts, depending on available resources and competing national health priorities.

**Table 1:** Surveillance and monitoring of HIV-exposed uninfected children in selected countries

	Current HIV Perinatal registry and surveillance linkage systems			Key research cohorts		
			
Country	Name/type	Information collected	Length of follow-up	Name	Length of follow-up	Current national recommendations
United States	None nationally currently; state-dependent Perinatal HIV Exposure Reporting Programs; Previous Enhanced Perinatal Surveillance (EPS) Program ended 2011; some state-dependent linkage to state cancer registries	State-Dependent; Prenatal & Intrapartum Data; Postnatal Data limited to infection status, postnatal ARVs, death, birth defect outcomes; New Jersey state with programme linking to state cancer registry	12–18 months for EPS; Up to 16 years for state linkage programme to cancer registry	PHACS (SMARTT) PACTG/ IMPAACT 219/219c	≥4 years	“Follow-up of children with exposure to ARVs should continue into adulthood because of the theoretical concerns regarding the potential for carcinogenicity of nucleoside analogue ARV drugs. Long-term follow-up should include annual physical examinations of all children exposed to ARV drugs. Innovative methods are needed to provide follow-up of infants, children, and youth with in utero exposure to ARV drugs. Information regarding such exposure should be part of ongoing permanent medical records for children, particularly those who are uninfected.” (DHHS Panel on Treatment of HIV-Infected Pregnant Women and Prevention of Perinatal Transmission, 2014)
Canada	Canadian Perinatal HIV Surveillance Program	Prenatal & Intrapartum Data; Postnatal Data limited to infection status, postnatal ARVs, death, major birth defects	18 months	CARMA, CMIS	CARMA (range up to 15 years); CMIS (2 years)	“Long-term follow-up and annual physical examinations, into adulthood, of HIV-uninfected infants exposed in utero and perinatally to antiretroviral medications is now recommended by the DHHS because of the potential carcinogenicity of the nucleoside analogs. Finally it is important to ensure continued psychosocial support for HIV-exposed uninfected children and their families.” (Prevention of Vertical HIV Transmission and Management of the HIV-exposed infant in Canada in 2014, CPARG & ID-SOCG)
France	Surveillance programme linking EPF and French National Cancer Registry	Anonymous linkage system between EPF and French National Cancer Registry	Up to 15 years	EPF	18–24 months	“If an HIV-exposed uninfected infant is asymptomatic, follow-up ends at 18–24 months. Follow-up should continue as necessary for unexplained symptoms, particularly neurological symptoms. There is, to date, no active program for the long term follow-up of asymptomatic HIV-exposed uninfected infants. Long term follow-up of symptomatic children may be justified and should be guided by best clinical practices. Families should alert the child's physician and/or the physician who treated the child during the first months of life of any significant clinical events.” (Medical Management of Persons Living with HIV, Report from Expert Panel of CNS and ANRS, 2013)
England	NHSPC; Flagging system to link NSHPC and death or cancer events in the national Health and Social Care Information Center (HSCIC)	Prenatal & Intrapartum Data; Postnatal Data limited to infection status, postnatal ARVs, death, and cancer events	Until HIV-non-infection documented (Range between 6 and 18 months) for NHSPC	CHART	–	“It is the responsibility of clinicians caring for women with HIV and their children to report them prospectively to the NSHPC. Aggregated data tables from the UK and Ireland of antiretroviral exposure and congenital malformations are regularly sent to the Antiretroviral Pregnancy Registry. Individual prospective reports should also be made to the Antiretroviral Pregnancy Registry antenatally with post-natal follow-up.” (Management of HIV Infection in Pregnant Women, 2014 interim update, British HIV Association)
Spain	None	–	–	NENEXP	18 months	“… the potential long-term toxicity in healthy exposed infants and the continuing emergence of new ARVs make it advisable to devise a mechanism whereby the identification and registration of potential adverse long-term effects of such exposures may be recorded. The Spanish Society of Pediatric Infectious Diseases recommends the creation of an anonymous national database supported by health authorities for this purpose. This database would require informed consent of the legal guardian of the infant (and the patient's own later) prior to inclusion in it. Unfortunately, these recommendations are in contrast with the reality of current practices in Spain where some specialized centers end monitoring of these patients at the time of seronegativity, others at 5 years of age, and still others follow throughout childhood.” (Recommendations by the Spanish Society of Pediatric Infectious Diseases for the follow-up of the child exposed to HIV and to ARV drugs during pregnancy and the neonatal period, 2012)
South Africa	New national registry beginning in 3 provinces including Western Cape	Basic perinatal and postnatal data, infection status, growth, TB symptom screening, developmental milestones assessment, significant events	To be defined	CDC-funded PMTCT Study	18 months	“Ideally all mothers and their infants should receive health care at the same consultation regardless of service point. The mother should understand the treatment and follow-up plan for herself and her infant. The RTHB should be completed prior to discharge after delivery, including recording HIV treatment/prophylaxis interventions received by mother during pregnancy, maternal illnesses, infant HIV prophylaxis and intended feeding method. The 1st postnatal visit is scheduled for day 3 but should take place within 6 days of life at the health facility.” Scheduled visits for infant follow-up should occur at 6, 10, and 14 weeks, monthly after 14 weeks until again at 6, 9, 12, and 18 months. (The South African Antiretroviral Treatment Guidelines 2013 – PMTCT Guidelines: Revised March 2013)
Thailand	National Surveillance Program of the Thai Ministry of Public Health	Prenatal & Intrapartum Data; Postnatal Data limited to infection status, postnatal ARVs, death	12–18 months	–	–	“The goals of the program are to reduce MTCT, provide health promotion for infants born to HIV-infected mothers, and provide appropriate medical treatment for parents in order to reduce the risk of infants or children being orphaned. Comprehensive care for HIV-infected women and family includes the following services: 1) Standard postpartum care should be provided, 2) General health promotion, e.g. nutritional support and exercise, should also be provided. 3) All postpartum women should be referred to internists for standard HIV treatment and care. Psychological and social supports needed for HIV-infected families may include the management of postpartum depression, psychosocial support for child rearing, and long-term family care.” (Thai National Guidelines for the Prevention of Mother-to-Child Transmission of HIV: March 2010)

ANRS=Agence nationale de recherches sur le SIDA et les hépatites virales; ARV=antiretroviral; CARMA=Canadian Institutes of Health Research Team Grant on HIV Therapy and Aging; CDC=Centres for Disease Control and Prevention; CHIPS=Collaborative HIV Paediatric Study; CMIS=Centre maternel et infantile sur le SIDA (Canadian Maternal Child Cohort); CNS=Conseil National du SIDA; CPARG=Canadian Paediatric and Perinatal AIDS Research Group; DHHS=Department of Health and Human Services; EPF=Enquête Périnatale Française (French Perinatal Cohort); HEU=HIV-exposed uninfected; ID-SOCG=Infectious Disease Committee of the Society of Obstetricians and Gynaecologists of Canada; NENEXP=Estudio clinico-epidemiologico de las parejas madre-hijo expuestas al VIH y/o a los fármacos antiretrovirales (Spanish Perinatal Cohort Study); NSHPC=National Study of HIV in Pregnancy and Childhood; PHACS=Pediatric HIV/AIDS Cohort Study; PMTCT=Prevention of Mother To Child Transmission; RTBH=Road-To-Health-Booklet; SMARTT=Surveillance Monitoring for ART Toxicities Study in HIV-uninfected Children Born to HIV-infected Women.

The U.S. Department of Health and Human Services recommends that HEU children be followed into adulthood due to the potential for carcinogenicity from nucleoside analogue ARVs [14]; Canadian guidelines mirror this and appeal for the psychosocial support of HEU children [15]. US guidelines also acknowledge a need for “innovative methods” to provide follow-up of these children and encourage that information regarding *in utero* HIV/ARV exposure be “part of ongoing permanent medical records for children.” In addition to several HEU research cohorts in both countries, the United States also recently reported a linking system in one state to match subjects from the Perinatal HIV Surveillance database and the state's cancer registry to monitor malignancy risk in HEU children [16]. A similar linkage system had been developed earlier in France, where the national cancer registry was linked in an anonymized fashion [17,18] to the major research cohort with longitudinal monitoring of HEU infants until 18 to 24 months [19–21]. The UK also has a national surveillance system of HIV-infected pregnant women and their infants (National Study of HIV in Pregnancy and Childhood, or NSHPC), which follows HEU children up to 18 months. National death and cancer event data in the UK have, in turn, been linked to data in the NSHPC to monitor death and cancer rates in HEU children [22,23]. In more resource-constrained settings, such as South Africa and Thailand, national guidelines recommend routine follow-up of HEU infants until approximately 18 months [24,25]. A South African pregnancy and HEU surveillance registry is being launched, which will ultimately include three provinces – KwaZulu-Natal, Gauteng and the Western Cape.

The differences in national guidelines on HEU longitudinal monitoring may be attributed to the differences in healthcare and research resources between countries. Regrettably, areas where high numbers of HIV/ARV-exposed pregnancies occur are also areas where healthcare, research and public health resources may be the most constrained. Despite the lack of consensus on the type of monitoring which HEU children merit, there does appear to be general agreement that some form of follow-up of HEU children is warranted [26] for the following reasons: 1) The type and timing of ARV exposures continue to evolve, at times outpacing research, making continued surveillance essential, 2) There are still many unknowns regarding long-term effects of this exposure. Given this, we now outline arguments in favour of and against disclosing perinatal HIV/ARV exposure status.

### The case for disclosure

The key arguments in favour of disclosure revolve around the assumption that there are substantial benefits (psychosocial and physical) for the child, HIV-infected mother and even other family members. In addition, disclosure may facilitate the conduct of large prospective HEU research cohorts in long-term monitoring, ultimately serving a critical public health function (Table 2).

**Table 2:** Major arguments for and against disclosure

Domain of consideration	For disclosure	Against disclosure
Psychosocial	Improves psychological health of mother and child	Worsens psychological health of mother and child – increase stigma and create increased stressors on an already fragile family environment
	Aids in transition and autonomy from childhood to adulthood healthcare	Creates a layer of unnecessary complexity during a time of transition when the adolescent may not be prepared to properly understand this exposure
Physical Health	Averts potential physical harm from long-term complications; early signals presented in current data are enough to warrant disclosure	Largely reassuring evidence that no physical harm with major early outcomes; not enough evidence of harm to see a benefit
Research/Public Health	Improves ability to continue long-term monitoring of more detailed outcomes in prospective research cohorts	Minimal ability to sustain long-term prospective HEU cohorts in the majority of the world. Surveillance programmes with linkage systems for the monitoring of major events in place and does not require disclosure

#### Psychosocial considerations

Despite the paucity of literature describing the impact of disclosure of a child's perinatal HIV/ARV exposure, several studies suggest positive effects on family relationships when disclosure of maternal HIV status to children occurs [11,27,28]. The Amagugu study in South Africa reported significant reduction in parental stress and child emotional/behavioural problems after an intervention to aid in disclosure of maternal HIV status [27]. In addition to higher family cohesion [29], United States studies have demonstrated lower levels of aggressiveness, poor self-esteem [11] and problem behaviours [28] in children whose mothers had disclosed compared to those who had not.

#### Physical health considerations

Clear physical harms from intrauterine HIV/ARV exposure would necessitate disclosure to the HEU individual. Several scientific arguments may be made to demonstrate current concerns for physical harms which may exist as a result of the exposure. First, developing theories on the origins of disease have suggested that foetal programming and the *in utero* milieu have a durable effect on the long-term health of an individual [30]. The *in utero* period represents a critical window during which changes may alter the biological setting of a foetus, thus placing the foetus at risk for future disease well into adulthood. For example, direct intrauterine toxins have the capacity to cause harmful effects even decades after the initial exposure, as in the case of antenatal diethylstilboestrol exposure and the increased risk of cervical, vaginal and breast cancer as well as infertility in adulthood [31]. Furthermore, *in utero* effects may present much later in life [32], such as with increased schizophrenia risk from maternal influenza and toxoplasmosis during pregnancy [33,34] or adult insulin resistance and cardiovascular disease from intrauterine growth restriction [35]. These long-term effects on neurobiological and metabolic pathways may not present with clear disease early in life but as the individual progresses through life and other adult exposures increase, there may be an accumulation of risk along the life spectrum, which places pressure on the programming a foetus may have undergone *in utero*, thereby increasing the risk of chronic diseases in adulthood [32]. Therefore, to avert the potential for major physical harm such as in the case of diethylstilboestrol exposure, disclosure is necessary in order to properly monitor HEU individuals into adulthood. Second, one could argue that in addition to childhood malignancies [16,17,23,36,37], there are a myriad of concerning data already surrounding malignancies as well as the mitochondrial [38–44], mental [45–47], bone [48–51], cardiovascular [52–54] and metabolic [55–57] health in HEU children as described herein.

##### Malignancy

Though some studies with less follow-up time have reported low cancer incidence rates, which have not exceeded population norms [16,23,36,37], the French EPF recently reported 10 cases of cancer in 53,052 person-years of follow-up as well as an increased risk (hazard ratio (HR)=13.6, 95% CI: 2.5–73.9) associated with didanosine (ddI)+3TC containing regimens versus zidovudine (AZT) monotherapy in HEU children [18]. In a subsequent study with an extended 153,939 person-years of follow-up of HEU children born between 1984 and 2014, the same group reported no differences in the incidence of cancer amongst HEU children compared to the general population but an increased risk with exposure to first trimester ddI (HR=5.5, 95% CI: 2.1–14.4) [17].

##### Mitochondrial toxicity

In France, combination ARVs compared to AZT monotherapy have been found to be associated with mitochondrial dysfunction (relative risk (RR)=2.5, 95% CI: 1.0–6.5, *p*=0.046), and several infants have shown clinical symptomatology [38,39]. Other studies have shown increased mitochondrial DNA in both AZT-exposed versus -unexposed [40,41] as well as HIV/ART-exposed versus -unexposed infants [42,43]. Aberrant mitochondrial morphology has also been demonstrated in infants exposed to *in utero* HIV/ART [44]. What remains unanswered is if and when these early mitochondrial effects translate into poor long-term health outcomes.

##### Mental health

A US study of HEU and perinatally HIV-infected children observed a higher prevalence of mental health problems in HEU children (38% vs. 25%, *p*=0.01) in unadjusted analyses [45]. In the U.S. Child and Adolescent Self-Awareness and Health study of perinatally HIV-infected and HEU youth, both groups exhibited high rates of any psychiatric disorder (49% in HEU youth) [46], and during the one to two years of follow-up, this rate did not decrease (57% at baseline to 54% later) in HEU youth [47].

##### Bone health

Pregnant rhesus macaques have shown compromised intrauterine growth and decreased foetal bone porosity in infants born to high-dose tenofovir (TDF)-treated SIV-infected and -uninfected monkeys [50,51]. The Pediatric HIV/AIDS Cohort Study (PHACS) reported decreased bone mineral content (BMC) in US newborns exposed to antenatal TDF [49]. In addition, the IMPAACT 1084 sub-study of Promoting Maternal and Infant Survival Everywhere (PROMISE) study found that both TDF/emcitritabine/lopinavir/ritonavir (*p*<0.001) as well as AZT/lamivudine (3TC)/lopinavir/ritonavir-exposed (*p*=0.002) infants showed lower BMC compared to those exposed to AZT monotherapy [48].

##### Cardiovascular and metabolic health

Recent studies have shown decreased left ventricular mass index and early diastolic annular velocity in HIV/ARV-exposed versus -unexposed infants [52]. In addition, increased risk of elevated cardiac troponin T in abacavir-exposed infants (OR=2.33, 95% CI: 1.03–5.26) and decreased risk of elevated N-terminal pro-brain natriuretic peptide in stavudine-exposed infants (OR=0.13, 95% CI: 0.02–0.99) have been reported [58], the long-term significance of either of which remains unclear. Lastly, studies have shown acylcarnitine and amino acid analytes, products of intermediary metabolism, were increased in ARV-exposed infants (43% vs. 0%, *p*=0.02) [57] as well as lower insulin levels and abnormal fuel substrate utilization in HEU infants at six weeks of life [56], which may affect the long-term metabolic health of HEU children.

#### Research/Public health considerations

Though they may not be feasible in all settings, prospective cohort studies can provide detailed, closely monitored, and well-described long-term outcomes data on HEU children. In order to continue these studies, it is ethically necessary to consent HEU individuals when they turn 18 since the HEU individual may have been an infant/child at enrolment when original consent was provided by a parent. This re-consenting in adolescence would require disclosure of the child's HEU status.

### The case against disclosure

The central argument against disclosure is that the harms of disclosure (psychological stress to the mother and child, the need to maintain privacy of the mother's HIV diagnosis, etc.) are greater than any benefit that might occur, or more simply, that there is no benefit due to the fact that no substantial health risks from intrauterine HIV/ARV exposure have been identified. Cumulative evidence strongly supports the continued use of ARVs in pregnancy, and data surrounding harmful HEU child outcomes are reassuring.

#### Psychosocial considerations

Though several studies discussed above have indicated psychosocial benefits to the mother and child from disclosure of maternal HIV status, there are almost an equal number citing worsening psychosocial functioning in children of mothers who disclose compared to those whose mothers do not [5,59–63]. This increased stressor on an already fragile household environment may produce enough psychosocial harm to argue against disclosure. Lower emotional and social functioning [59] as well as increased externalizing behavioural problems [5] have been reported in cross-sectional studies of children whose mothers disclosed. Adolescents whose mothers disclosed may appear to be at risk for early parentification out of a felt need to support their HIV-infected mother [60,64]. Other reports have shown that these adolescents reported higher rates of emotional distress [28,63], high-risk behaviours [63,65] and negative school performance [62] compared to adolescents whose mothers had not disclosed. Disclosure to HEU adolescents may create a layer of unnecessary complexity during a time when the adolescent may not be prepared to properly understand this exposure.

#### Physical health considerations

If the risk of physical harm from *in utero* HIV/ARV exposure is not substantial, it may be argued that disclosure is not necessary. What dictates “substantial” is debatable, but many consider outcomes involving birth weight [66–75], congenital defects [76–81], early neurodevelopment [82–84] and growth [70,75,85–87] as significant, and none of these have demonstrated a clear association with *in utero* HIV/ARV exposure (Table 3). Even pre-term birth, which has been shown in several studies to be associated with ART [74,89–92,95] is still an early infant outcome and would occur and be managed well before adolescence, the time of disclosure.

**Table 3:** Major studies assessing complications of *in utero* maternal HIV and antiretroviral exposure in HIV-exposed infants

Authors (reference)	Study subjects/cohort	Study design	Sample size	Age period studied	*In utero* exposure of interest	outcomes measured	Results
**Birth outcomes**							
*SGA/LBW*							
Habib *et al*. 2008 [88]	Tanzania	Cohort	14,444	Birth	ARV-/HIV+ vs. ARV-/HIV- ARV+/HIV+ vs. ARV-/HIV- Unknown maternal HIV status vs. ARV-/HIV-	SGA at 10th percentile	Increased risk SGA associated with: *ARV-/HIV+ vs. ARV-/HIV- (OR=1.64, 95% CI: 1.1–2.4) *HIV status unknown vs. ARV-/HIV- (OR=1.2, 95% CI: 1.1–1.4) Infants of treated HIV+ women with similar risk of SGA as infants of HIV- women
Sperling *et al*. 1998 [66]	US (PACTG 076)	RCT	342	Birth to 18 months	Antepartum-Intrapartum-Newborn AZT+ vs. AZT-	WAZ, LAZ, HCAZ SGA	No association between AZT and SGA
ECS. 1999 [67]	Europe (ECS)	Cohort	2274	Birth	AZT+ vs. AZT-	LBW (<2500 g)	Decreased risk of LBW associated with AZT+ (any) (OR=0.55, 95% CI: 0.39–0.79)
Chotpitayasunondh *et al*. 2001 [68]	Thailand	RCT	395	Birth to 18 months	AZT+ vs. AZT-	WAZ, LAZ, HCAZ	No differences in mean BW, birth length
Briand *et al*. 2006 [85]	Thailand (PHPT-1)	RCT	1408	Birth, 6 weeks, 18 months	AZT+ (≥7.5 weeks) vs. AZT+ (<7.5 k)	WAZ, LAZ, WLZ	Decreased birth WAZ, WLZ in AZT+ (>7.5 weeks)
Siberry *et al*. 2012 [69]	US (PHACS)	Cohort	2010	Birth to 1 year	TDF+ vs. TDF-	SGA, LBW WAZ, LAZ, HCAZ	No association between *in utero* TDF and LBW, SGA or birth LAZ and HCAZ
Ransom *et al*. 2013 [70]	US (IMPAACT 1025)	Cohort	2025	Birth to 6 months	TDF+ vs. TDF-	SGA, WAZ	No association between *in utero* TDF and SGA (OR=1.09, 95% CI: 0.77–1.52) No differences in birth WAZ (*p*=0.9)
Gibb *et al*. 2012 [71]	Uganda, Zimbabwe (DART)	RCT	182	Birth to 3 year	TDF+ vs. TDF-	LBW	No difference in rates of LBW between groups
Tuomala *et al*. 2002 [72]	US (PACTG 076 & 185, PACTS, WITS, & 3 single sites)	RCT & Cohort	3266	Birth	any ART vs. no ART cART vs.AZT monotherapy cART w/out PI vs. AZT monotherapy cART w/PI vs. AZT monotherapy cART w/PI vs. w/out PI	LBW, VLBW (<1500 g)	cART not associated with LBW Increased risk of VLBW associated with cART w/PI vs. w/out PI (OR=3.56, 95% CI: 1.04–12.19)
Szyld *et al*. 2006 [73]	Latin America & Caribbean (NISDI)	Cohort	681	Birth	PI- vs. NNRTI- vs. 1–2 NRTI-based cART	LBW	No increased risk of LBW (OR=1.5, 95% CI: 0.7–3.2 for PI; OR=0.6, 95% CI: 0.3–1.5 for NNRTI)
Watts *et al*. 2013 [74]	US (PHACS)	Cohort	1869	Birth	cART with PI vs. mono/dual therapy ART cART with NNRTI vs. mono/dual therapy ART cART with ≥3 NRTIs vs. mono/dual therapy ART cART initiated pre-pregnancy vs. cART initiated after 1^st^ trimester	SGA at 10th percentile	No association between SGA and cART
Nielsen-Saines *et al*. 2012 [75]	Africa, Thailand, India, Brazil (ACTG 5190/IMPAACT 1054)	Cohort	236	Birth to 18 months	cART vs. AZT (≥7 days) vs. AZT (intrapartum only)	SGA	No differences in SGA between groups
Chen *et al*. 2012 [89]	Botswana	Cohort	33,148	Birth	HIV+ vs. HIV- cART+ (initiated pre-pregnancy)/HIV+ vs. all other/HIV+ cART+ vs. AZT monotherapy cART+ (initiated pre-pregnancy) vs. cART+ (initiated during pregnancy)	SGA at 10th percentile	Increased risk of SGA associated with: *in utero HIV exposure (OR=1.8, 95% CI: 1.7–1.9) *cART+ (initiated pre-pregnancy)/HIV+ vs. all other/HIV+ (OR=1.8, 95% CI: 1.6–2.1) *in utero cART vs. AZT monotherapy (OR=1.5, 95% CI: 1.2–1.9) *in utero cART+ initiated pre-pregnancy vs. during pregnancy (OR:1.3, 95% CI: 1.0–1.5)
*Preterm birth*							
*Combination ART exposure*							
ECS. 2003 [76]	Europe (ECS)	Cohort	2414	Birth	cART vs. no ART vs. AZT monotherapy	Preterm birth	Increased risk preterm birth (OR=2.66, 95% CI: 1.52–4.67 for cART without PI; OR=4.14, 95% CI: 2.36–7.23 for cART with PI) with cART
Townsend *et al*. 2010 [90]	US, Europe (PSD, ECS, NSHPC)	Pooled analysis of registry & cohorts	19,585	Birth	cART vs. dual therapy cART	Preterm birth	Increased risk preterm birth (OR=1.49, 95% CI: 1.19–1.87) with cART vs. dual therapy cART
Chen *et al*. 2012 [88]	Botswana	Cohort	13,181	Birth	cART vs. AZT monotherapy pre-pregnancy cART initiation vs. all others	Preterm birth	Increased risk preterm birth with cART (OR=1.4, 95% CI: 1.2–1.8) and pre-pregnancy cART initiation (OR=1.2, 95% CI: 1.1–1.4)
Sibiude *et al*. 2012 [91]	France (EPF)	Cohort	1253	Birth	cART vs. AZT monotherapy Ritonavir boosted PI vs. non-ritonavir boosted PI	Preterm birth	Increased risk preterm birth with cART (OR=1.69, 95% CI: 1.38–2.07) Increased risk preterm birth with ritonavir boosted PI (OR=2.03; 95% CI: 1.06–3.89)
Short *et al*. 2014 [92]	UK	Cohort	331	Birth	cART vs. AZT monotherapy	Preterm birth	Increased risk preterm birth with cART (OR=5.0, 95% CI: 1.5–16.8)
Lopez *et al*. 2012 [93]	Spain	Matched cohort	1557	Birth	HIV+/ARV+ or ARV- vs. HIV- cART during 2^nd^ half of pregnancy vs. untreated	Preterm birth	Increased risk preterm birth with maternal HIV infection (ARV+/−) (OR=2.5, 95% CI: 1.5–3.9) Increased risk iatrogenic preterm birth with cART during 2^nd^ half of pregnancy (OR=6.16, 95% CI: 1.42–26.80)
*PI exposure*							
Cotter *et al*. 2006 [94]	US	Registry	1337	Birth	PI-based cART vs. non PI-based cART cART vs. AZT monotherapy Any ART vs. none	Preterm birth	Increased risk preterm birth (OR=1.8, 95% CI: 1.1–3.0) for PI vs. non PI-based cART No increased risk for preterm birth with cART vs. AZT monotherapy or ART vs. no ART
Schulte *et al*. 2007 [95]	US (PSD)	Registry	8793	Birth	PI-based cART vs. dual therapy ART No ART vs. dual therapy ART	Preterm birth	Increased risk preterm birth (OR=1.21, 95% CI: 1.04–1.40) Increased risk preterm birth (OR=1.16, 95% CI: 1.02–1.32)
Grosch-Woerner *et al*. 2008 [96]	Germany	Cohort	183	Birth	PI-based cART vs. AZT monotherapy	Preterm birth	Increased risk preterm birth (OR=3.4, 95% CI: 1.1–10.2) with PI-based cART
Szyld *et al*. 2006 [73]	Latin America and Caribbean (NISDI)	Cohort	681	Birth	PI- vs. NNRTI- vs. 1-2 NRTI-based ART	Preterm birth	No increased risk of preterm birth (OR=1.1, 95% CI: 0.5–2.8 for PI; OR=0.6, 95% CI: 0.2–1.7 for NNRTI)
Shapiro *et al*. 2010 [97]	Botswana	RCT	709	Birth	PI- vs. triple NRTI- vs. NNRTI- based ART	Preterm birth (secondary outcome)	Increased rate preterm birth in PI arm (23% vs. 15% vs. 10%)
Watts *et al*. 2013 [74]	US (PHACS)	Cohort	1869	Birth	1^st^ trimester PI vs. NNRTI vs. ≥3 NRTIs-based ART	Preterm birth	Increased risk preterm birth with 1st trimester PI (OR=1.55, 95% CI: 1.16–2.07)
**Congenital anomalies**							
ECS. 2003 [76]	Europe (ECS)	Cohort	2414	Birth	Any ART vs. no ART	Any congenital anomaly	Similar patterns and prevalence rates of congenital anomalies in ART vs. no ART exposure (1.4% vs. 1.6%, *p*=0.762)
Townsend *et al*. 2009 [77]	UK	Surveillance	8242	Birth	Late vs. early ART exposure PI- vs. NNRTI- vs. NRTI only- vs. 2 class-cART	Any congenital anomaly	Overall prevalence of congenital anomalies=2.2%, 95% CI: 2.5–3.2% No differences in congenital anomalies by timing or class of ART exposure
Ford *et al*. 2014 [98]	–	Pooled analysis	2026 (pooled overall prevalence) 11,325 (pooled RR)	Birth	EFV	Any congenital anomaly NTD	Overall prevalence of congenital anomalies=1.63%, 95% CI: 0.78–2.48% No differences in overall congenital anomalies between EFV vs. non-EFV ART; (RR=0.78, 95% CI: 0.56–1.08)
Watts *et al*. 2011 [78]	PACTG 316	Cohort	1408	Birth	Multiple ARVs	Any congenital anomaly	Overall prevalence of congenital anomalies=4.2%, 95% CI: 3.3–5.4%
Liu *et al*. 2014 [79]	South Africa Zambia	Cohort	600	Birth to 1 year	cART since conception	Any congenital anomaly	Overall prevalence of congenital anomalies=6.2%; Prevalence of major congenital anomalies=2.2%
Sibiude *et al*. 2014 [80]	France	Cohort	13,124	Birth to 18 months	Multiple ARVs	Any congenital anomaly as defined by EUROCAT and by MACDP	Overall prevalence of congenital anomalies=4.4%, 95% CI: 4.0–4.7% using EUROCAT
Knapp *et al*. 2012 [99]	IMPAACT 1025	Cohort	1112	Birth	EFV	Any congenital anomaly	Overall prevalence of congenital anomalies=5.5%, 95% CI: 4.22–6.99. Increased risk of congenital anomaly with 1st trimester EFV (OR=2.84, 95% CI: 1.13–7.16)
Antiretroviral Pregnancy Registry Executive Summary 2015 [100]	US (Antiretroviral Pregnancy Registry)	Registry	7135	Birth	Any 1^st^ trimester ART	Any congenital anomaly	Overall prevalence of congenital anomalies=2.8%, 95% CI: 2.5–3.3%
Williams *et al*. 2014 [101]	PHACS	Cohort	2580	Birth	Multiple ARVs	Any congenital anomaly	Overall prevalence of congenital anomalies=6.8%, 95% CI: 5.9–7.8%
**Endocrine/metabolic**							
*Infant/Child growth*							
ECS. 2005 [102]	Europe (ECS)	Cohort	1912	Birth to 18 months	cART vs. No/AZT monotherapy	WAZ, LAZ, HCAZ	Decreased WAZ [β=(−0.10), *p*=0.019], LAZ [β=(−0.12), *p*=0.008], and HCAZ [β=(−0.14), *p*=0.001] associated with cART
Briand *et al*. 2006 [85]	Thailand (PHPT-1)	RCT	1408	Birth, 6 weeks, 18 months	AZT+ (≥7.5 weeks) vs. AZT+ (<7.5 weeks)	WAZ, LAZ, WLZ	No differences in 6 weeks or 18 months WAZ, LAZ, WLZ between groups
Ibieta *et al*. 2009 [86]	Spain (FIPSE)	Cohort	601	Birth to 2 years	ARV+/HIV+ vs. ARV-/HIV- ARV-/HIV+ vs. ARV+/HIV+ cART w/PI+ vs. cART w/out PI	WAZ, LAZ, HCAZ	No differences in WAZ, LAZ, HCAZ between groups
Nielsen-Saines *et al*. 2012 [75]	US (ACTG 5190) Africa, Thailand, India, Brazil (IMPAACT 1054)	Cohort	236	Birth to 18 months	cART vs. AZT (≥7 days) vs. AZT (intrapartum only)	WAZ, LAZ, HCAZ	No differences in WAZ, LAZ, HCAZ by ARV exposure
Siberry *et al*. 2012 [69]	US (PHACS)	Cohort	2010	Birth, 1 year	TDF+ vs. TDF-	WAZ, LAZ, HCAZ	Decreased LAZ [ß=(−0.17) vs. (−0.03), *p*=0.04] and HCAZ (ß=0.17 vs.0.42, *p*=0.02) at 1 year associated with *in utero* TDF
Neri *et al*. 2013 [87]	US	Matched case control	111	Birth to 2 year	cART+/HIV+ vs. matched cART-/HIV- cART+/HIV+ vs. NHANES	WAZ, WLZ	No differences in growth between HEU and HIV-unexposed infants
Ransom *et al*. 2013 [70]	US (IMPAACT 1025)	Cohort	2025	Birth, 6 months	TDF+ vs. TDF-	WAZ	No differences in WAZ at 6 months between groups
*Mitochondrial toxicity*							
Perinatal Safety Review Working Group. 2000 [103]	US (PACTG 076 & 185, WITS, PACTS, PSD, PHS)	Cohort	23,265	Birth to <60 months	AZT monotherapy AZT-3TC Other	Mortality from mitochondrial dysfunction	No deaths or associated signs/symptoms suggestive of or proven to result from mitochondrial dysfunction
Barrett *et al*. 2003 [39]	France	Cohort	4426	Birth to 18 months	ART (any)+/HIV+ vs. ART-/HIV+	Mitochondrial dysfunction classified as: *Established* (compatible clinical symptoms + Decrease in OXPHOS or Abnormal mt morphology) vs. *Possible* (compatible clinical symptoms + hyperlactatemia or minor mt morphologic abnormalities)	12 subjects with “Established” mt dysfunction; 14 with “Possible” mitochondrial dysfunction Increased incidence of mitochondrial dysfunction Combination NRTIs (vs. AZT monotherapy) associated with increased risk of mitochondrial dysfunction (RR=2.5, 95% CI: 1.0–6.5, *p*=0.046)
Aldrovandi *et al*. 2010 [40]	US (WITS, PACTG 1009)	Cohort	624	Birth to 5 year	AZT-3TC+/HIV+ vs. AZT+/HIV+ vs. AZT-/HIV+ vs. AZT-/HIV-	Mitochondrial DNA content	Decreased mitochondrial DNA levels (AZT or AZT-3TC+/HIV+ vs. AZT-/HIV-) Increased mitochondrial DNA (AZT+/HIV+ vs. AZT-/HIV+ & AZT-3TC+/HIV+ vs. AZT+/HIV+)
Brogly *et al*. 2010 [104]	US (IMPAACT)	Cohort	982	Birth to 1 year	Any ART+ vs. ART- Any NRTI+ vs. NRTI- 3TC, AZT, ABC, d4T, ddI, & TDF individually	Possible mitochondrial dysfunction defined as compatible clinical signs using EPF definition	3 subjects with possible mt dysfunction No association between ART and mitochondrial dysfunction
McComsey *et al*. 2008 [42]	US (ACTG 5084)	Cohort	136	Birth	cART+/HIV+ vs. cART-/HIV-	Mitochondrial DNA content; Respiratory chain activity	Increased mitochondrial DNA levels No difference in Complex II:IV ratio
Côté *et al*. 2008 [43]	Canada	Cohort	154	Birth to 6 months	cART+/HIV+ vs. cART-/HIV-	Mitochondrial DNA content	Increased mitochondrial DNA levels
Kunz *et al*. 2012 [41]	Tanzania	Cohort	83	Birth	AZT+/sdNVP+/HIV+ vs. AZT-/sdNVP+/HIV+	Mitochondrial DNA content; Mitochondrial deletion dmtDNA4977	Increased mitochondrial DNA levels No deletion of dmtDNA4977
*Intermediary Metabolism*							
Kirmse *et al*. 2013 [57]	US	State Registry	2371	Birth	HIV+/ARV+ vs. HIV-/ARV-	Abnormal newborn metabolic screen and acylcarnitine profiles	Increased rate of abnormal newborn metabolic screen in HIV-exposed infants compared to general population (2.2 vs. 1.2%, *p*=0.0003); Increased frequency of abnormal acylcarnitine profiles (43 vs. 0%, *p*=0.02)
Jao *et al*. 2015 [56]	Cameroon	Cohort	366	Birth to 6 weeks	HIV+/ARV+ vs. HIV-/ARV- Postnatal AZT HEU vs. Postnatal NVP HEU vs. HUU	Pre-prandial infant insulin and HOMA-IR Acylcarnitines and branched-chain amino acids	Lower pre-prandial insulin in postnatal AZT HEU vs. HUU infants (β: −0.116, *p*=0.012) and in postnatal NVP HEU vs. HUU infants (β: −0.070, *p*=0.022)
*Bone Health*							
Vigano *et al*. 2011 [105]	Italy	Cohort	68	Birth to 6 year	TDF+ vs. TDF-	Tibial SOS via ultrasound Bone markers	No differences in tibial SOS No differences in bone markers
Mora *et al*. 2012 [106]	Italy	Cohort	131	Birth, 4 months, 12 months	ARV+/HIV+ vs. ARV-/HIV-	Tibial SOS via ultrasound Bone markers	No differences in tibial SOS No differences in bone markers
Siberry *et al*. 2015 [49]	US (PHACS)	Cohort substudy	143	Birth to 1 month	TDF+ vs. TDF-	BMC via bone DXA	Mean BMC decreased in TDF-exposed infants 56.0 vs. 63.8g *p*=0.002)
Siberry *et al*. 2016 [48]	Multi-national (IMPAACT PROMISE 1084 substudy)	RCT substudy	362	Birth to 21 days of life	AZT monotherapy vs. TDF/FTC/Lop/r vs. AZT/3TC/Lop/r	Whole body BMC via bone DXA	Lower whole body BMC in: TDF/FTC/Lop/r vs. AZT monotherapy (*p*<0.001) AZT/3TC/Lop/r vs. AZT monotherapy (*p*=0.002) No difference between TDF/FTC/Lop/r vs. AZT/3TC/Lop/r arms
**Cardiovascular**							
Lipschultz *et al*. 2000 [107]	US (PHACS)	Cohort	611	Birth to 15 months	Continuous AZT+/HIV+ vs. AZT-/HIV+	Cardiac structure and function via echo	No differences in cardiac structure or left ventricular function
Cade *et al*. 2012 [52]	US	Matched cohort	60	8 to 12 year olds	ARV+/HIV+ vs. ARV-/HIV-	LV EDV LV mass	Decreased LV mass index and early diastolic annular velocity in HIV/ARV-exposed children
Wilkinson *et al*. 2013 [58]	US (PHACS)	Cohort	338	Birth to 5 year	Specific ARVs	Cardiac biomarkers: hsCRP, cTnT, NT-proBNP	Increased risk of elevated cTnT levels in ABC-exposed infants (OR=2.33, 95% CI: 1.03–5.26) Decreased risk of elevated NT-proBNP in d4T-exposed infants (OR=0.13, 95% CI: 0.02–0.99)
**Neurodevelopmental/mental health**							
Williams *et al*. 2010 [82]	US (PACTG 219)	Cohort	1840	Birth to 2 year	ARV+/HIV+ vs. ARV-/HIV+	MDI & PDI scores from Bayley Scales of Infant Development	No differences in MDI or PDI scores
Sirois *et al*. 2013 [83]	US (PHACS)	Cohort	374	Birth to 15 months	cART+/HIV+ vs. no ART/HIV+ cAtRT+/HIV+ vs. AZT monotherapy/HIV+ PI- vs. NNRTI- vs. NRTI only based cART	Bayley Scales of Infant Development Version III	No differences in mean scores for any of the 5 domains within Bayley III
Kerr *et al*. 2014 [108]	Thailand, Cambodia	Cohort	333	Mean age 7.6 years	ART+/HIV+ vs. ARV-/HIV-	Wechsler Intelligence Scale; Stanford Binet II Memory Tests	Verbal IQ: Adjusted mean difference =−6.13, *p*=0.004 Full Scale IQ: Adjusted mean difference =−4.57, *p*=0.03 Stanford Binet Bead Memory: Adjusted mean difference =−3.72, *p*=0.01
Nozyce *et al*. 2014 [84]	US (PHACS)	Cohort	739	5 to 13 year olds	PI-based cART vs. NNRTI-based cART vs. non-cART regimen vs. no ARV	WPPSI-III (5 year old) WASI (7, 9, 11 and 13 year old) WIAT-II-A	No associations between any ARV regimen/class and cognitive or academic outcomes
Malee *et al*. 2011 [45]	US (PHACS)	Cohort	416 total (121 HEU)	55% less than 12 years old	Perinatally HIV-infected and HEU youth	Mental Health problems using BASC-2 Self-Report of Personality and BASC-2 Parent Rating Scale	Rates of mental health problems higher in HEU vs. perinatally HIV-infected youth (38% vs. 25%, *p*=0.01)
Mellins *et al*. 2012 [47]	US (CASAH)	Cohort	340 total (134 HEU)	Mean age 12.2 years (SD=2.3)	Perinatally HIV-infected and HEU youth	Psychiatric diagnoses using DISC-IV	High rates of overall psychiatric disorders in HEU youth (49%) No change in these rates over longitudinal follow-up (mean 18.5 years follow-up)
**Oncologic**							
Hanson *et al*. 1999 [37]	US (PACTG 076 & 219, WITS)	Cohort	727	Range: [Birth-1 month] – [Birth-6 years]	AZT	Any malignancy	Overall RR of tumour=0.0, 95% CI: 0–17.6
Brogly *et al*. 2006 [36]	US (PACTG 219)	Cohort	2077	Not reported	Multiple regimens	Any malignancy	One incident of cancer in 7871 person years of follow-up (incidence rate=0.127 per 1000 person-years, 95% CI: 0.003–0.708)
Hankin *et al*. 2007 [23]	UK (NSHPC)	Surveillance	2612	Not reported	Multiple regimens	Any malignancy	No cases of cancer over 6593 child-years of follow-up
Benhammou *et al*. 2008 [18]	France (EPF)	Cohort	9127	53,052 person years follow-up	Multiple regimens	Any malignancy	10 cases of cancer in 53,052 person-years of follow-up Increased risk of cancer (HR=13.6, 95% CI: 2.5–73.9) with ddI+3TC containing regimens vs. AZT monotherapy
Hleyhel *et al*. 2016 [17]	France (EPF)	Cohort	15,163	153,939 person years follow-up	Multiple regimens	Any malignancy	21 cases of cancer in 153,939 person years of follow-up No difference in cancer incidence amongst HEU vs. general population Increased risk of cancer (HR=2.5, 95% CI: 1.01–5.19) with ddI exposure and significantly increased risk with 1st trimester ddI (HR=5.5, 95% CI: 2.1–14.4)
Ivy *et al*. 2015 [16]	US	State registry/surveillance	3087	1 to 16 years	Multiple regimens	Any malignancy	4 cases of cancer in 3087 HIV-exposed children (29,099 person years) between 1995 and 2010; 13.7 per 100,000 person years cancer incidence rate (95% CI: 3.7–35.2)

3TC=lamivudine; ABC=abacavir; ART=antiretroviral therapy; ARV=antiretroviral; AZT=zidovudine; cART=combination antiretroviral therapy; BASC-2=Behavior Assessment System for Children, 2nd edition; CASAH=Child and Adolescent Self-Awareness and Health; CI=Confidence Interval; cTnT=cardiac Troponin T; d4T=stavudine; DART=Development of AntiRetroviral Therapy in Africa; ddI=didanosine; DISC-IV=Diagnostic Interview Schedule for Children; DXA=Dual Energy X Ray Absorptiometry; ECS=European Collaborative Study; EDV=end diastolic volume; EFV=efavirenz; EPF=Enquête Périnatale Française; EUROCAT=European Surveillance of Congenital Anomalies; FIPSE=Fundación para la Investigación y la Prevención del Sida en Espana; HCAZ=Head Circumference for Age z score; HEU=HIV-exposed uninfected; HOMA-IR=Homeostatic Model Assessment-Insulin Resistance; HR=Hazard Ratio; hsCRP=high sensitivity C-reactive Protein; HUU=HIV-unexposed uninfected; IMPAACT=International Maternal Pediatric Adolescent AIDS Clinical Trials Group; LAZ=Length for Age z score; LBW=low birth weight; LV=left ventricular; MACDP=Metropolitan Atlanta Congenital Defects Program; MDI=Mental Developmental Index; NHANES=National Health and Nutrition Examination Survey; NSHPC=National Study of HIV in Pregnancy and Childhood; NISDI=National Institute of Child Health and Human Development International Site Development Initiative; NNRTI=non-nucleoside reverse transcriptase inhibitor; NRTI=nucleoside reverse transcriptase inhibitor; NTD=neural tube defect; NT-proBNP=N-terminal pro-brain natriuretic peptide; OR=odds ratio; PACTG=Pediatric AIDS Clinical Trials Group; PDI=Psychomotor Developmental Index; PHACS=Pediatric HIV/AIDS Cohort Study; PHPT-1=Perinatal HIV Prevention Trial-1; PHS=Pediatric HIV Surveillance; PI=protease inhibitor; PROMISE=Promoting Maternal and Infant Survival Everywhere; PSD=Pediatric Spectrum of HIV Disease Project; RCT=randomized controlled trial; RR=relative risk; sdNVP=single dose nevirapine; SD=standard deviation; SGA=small-for-gestational age; SOS=speed of sound; TDF=Tenofovir disoproxil fumarate; UK=United Kingdom; US=United States; VLBW=very low birth weight; WASI=Wechsler Abbreviated Scale of Intelligence; WAZ=weight for age z score; WIAT-II-A=Wechsler Individual Achievement Test – Version II Abbreviated; WITS=Women and Infants Transmission Study; WLZ=weight for length z score; WPPSI-III=Wechsler Preschool & Primary Scale of Intelligence-Version III

##### Birth weight

Despite one large study in Botswana which reported an increased risk for small-for-gestational age (SGA)outcomes in HEU infants [88] (odds ratio (OR) for SGA=1.5, 95% CI: 1.2–1.9), the vast majority of reports have not found a consistent association between *in utero* HIV/ARVs and low birth weight (LBW) or SGA outcomes. Exposure to antenatal AZT has not been found to be associated with SGA in the United States [66] or LBW in Europe [67]. In addition, studies in the United States [72,74] and another multi-country study [75] have reported no associations between antenatal ART and LBW/SGA. A large study in Latin America also did not find risks for LBW when comparing classes of ARVs [73]. Lastly, two US studies [69,70] did not find increased risks for SGA, and one Ugandan study did not find increased risks for LBW [71] with intrauterine TDF exposure.

##### Congenital defects

In general, there has not been evidence for an increased rate of birth defects (overall rates 1.4–6.2%) associated with HIV/ARV exposure [76–81,98,99]. The two largest surveillance registries for congenital anomalies in the UK [77] and the United States [100] have found low rates of birth defects consistent with other cohorts in Europe [76] and the United States [78]. Few reports have emerged from low-income countries, but one pilot ART registry from South Africa and Zambia identified a 6.2% prevalence rate for all and 2.2% for major congenital anomalies [79]. Despite earlier reports in humans revealing neural tube defects in infants exposed to efavirenz (EFV) early in gestation [99,109], a more recent meta-analysis of 2026 infants countered these results and found no risk (RR=0.78, 95% CI: 0.56–1.08) [93]. In addition, the French EPF [80] and a recent US study [101] found overall low rates of congenital anomalies associated with EFV.

##### Neurodevelopment

The Pediatric AIDS Clinical Trial Group (PACTG) 219 noted no differences in mental or psychomotor developments in 1840 HIV/ARV-exposed versus -unexposed children [82]. Similar findings were found in the PHACS cohort when assessing the effects of ART exposure as well as differing ART class regimens [83]. A more recent study within PHACS also evaluated cognitive outcomes in older HEU children and did not find associations between any perinatal ART class regimens and cognitive and academic scores [84]. One study from Thailand reported small reductions in Wechsler Intelligence Scale testing comparing HEU to HUU children but acknowledged the uncertainty around the long-term clinical significance of these findings [108].

##### Growth

With the exception of a few studies [69,102], most large studies have not reported problems with early postnatal growth after *in utero* HIV/ARV exposure [70,75,85–87]. A Thai study found no differences in weight-for-age, weight-for-length, or length-for-age z scores between infants exposed to <7.5 versus ≥7.5 weeks of AZT [85]. A Spanish cohort reported similar findings when evaluating HIV/ARV-exposed versus HIV-unexposed infants and HIV- versus HIV/ARV-exposed infants [86]. A multi-national study had comparable results when examining ART versus AZT monotherapy exposure [75].

#### Research/public health considerations

While large prospective research cohorts may be the most comprehensive method to monitor HEU children long-term, they require re-consenting an HEU adolescent and disclosure of HEU status, rendering the feasibility and sustainability of these in all settings challenging. Surveillance programmes with linkage systems for the monitoring of major events may be used instead for long-term monitoring, particularly in resource-constrained settings where the largest proportion of HEU children reside, and would not require disclosure in most circumstances.

## Conclusions: to disclose or not to disclose

As we confront the many unknowns outlined above – the continued high rates of HIV infection in women globally with an increasing and aging population of HEU children – the tensions surrounding disclosure will need to be considered carefully. Clinicians in both high- and low-resource settings face the difficulty of balancing the need to respect a mother's rights to privacy and prevent further familial psychosocial harm versus the potential benefits to the HEU adolescent and his/her family from disclosure of exposures [12]. In these settings, careful assessment (and re-assessments) of the risk/benefit ratio, the HEU individual's changing and maturing needs, and the mother's need for privacy should be considered during the discussion of whether to disclose or not. At present, it is not clear that we have sufficient evidence on whether long-term adverse effects are associated with *in utero* HIV/ARV exposures, making it difficult to mandate universal disclosure. If evidence for a particularly threatening complication from intrauterine HIV/ARV exposure unsurfaces through research, countries may grapple seriously with how best to manage and address this issue, particularly in areas where healthcare infrastructures are already fragile, or health literacy is low. Data on long-term reproductive health effects, immunologic dysfunction, risk of adult onset malignancies, cardiovascular disease, or neurodevelopmental and mental health disorders in adulthood are still inconclusive with no published reports in HEU adults. To meet this void of evidence, research and long-term monitoring likely needs to be continued, and there is general consensus among health professionals and parents of HEU children that more data need to be collected on the long-term health of HEU individuals [26]. Research methods using anonymized surveillance systems linked to other national registries will prove indispensable as data are gathered to understand whether *in utero* HIV/ARV exposure may result in long-term harm, but prospective research cohorts evaluating this question will need to contend with the need for disclosure to HEU individuals in order to continue long-term follow-up into adulthood – a conundrum where the rationale for the research clashes with the reasons for not mandating universal disclosure at present.

As more countries adopt electronic medical record (EMR) systems, the HEU status of an individual will be an important piece of the health record which will follow the infant not only through childhood and adolescence but also adulthood, which may cause disclosure to be a moot point once young adults access their records. With increasing understanding of the influence of early intrauterine exposures on long-term health outcomes, this practice of early and continued documentation should become the standard as EMR systems expand, potentially rendering disclosure an easier and more natural process for parents/caregivers. Permanent documentation via EMR of perinatal exposures may also improve research and surveillance/registry efforts which are required in order to continue monitoring into adulthood and ultimately gather essential data which are still lacking. Thus, clinicians and researchers should continue to approach the dialogue around mother–child disclosure with sensitivity, an understanding of maternal needs in addition to a child/adolescent's development and readiness to hear information, and a cogent consideration of the evolving risks and benefits as new information becomes available but work to maintain documentation of an individual's perinatal HIV/ARV exposures as a vital part of his/her medical records. As more long-term adult safety data on *in utero* HIV/ARV exposures become available, these decisions may become clearer, but for the moment, they remain complex and multi-faceted.
